# A novel upward-looking hydroacoustic method for improving pelagic fish surveys

**DOI:** 10.1038/s41598-017-04953-6

**Published:** 2017-07-06

**Authors:** Roman Baran, Tomáš Jůza, Michal Tušer, Helge Balk, Petr Blabolil, Martin Čech, Vladislav Draštík, Jaroslava Frouzová, Asanka D. Jayasinghe, Ievgen Koliada, Tomáš Mrkvička, Milan Muška, Daniel Ricard, Zuzana Sajdlová, Lukáš Vejřík, Jan Kubečka

**Affiliations:** 1 Institute of Hydrobiology, Biology centre of the Czech Academy of Sciences, České Budějovice, Czech Republic; 2 Faculty of Science, University of South Bohemia, České Budějovice, Czech Republic; 3 Department of Physics, University of Oslo, Oslo, Norway; 4 Department of Limnology, University of Ruhuna, Wellamadama, Matara Sri Lanka; 5 Faculty of Economics, University of South Bohemia, České Budějovice, Czech Republic; 6 Fisheries and Oceans Canada, Gulf Fisheries Centre, Moncton, Canada

## Abstract

For ethical reasons and animal welfare, it is becoming increasingly more important to carry out ecological surveys with a non-invasive approach. Information about fish distribution and abundance in the upper water column is often fundamental. However, this information is extremely hard to obtain using classical hydroacoustic methods. We developed a rigid frame system for pushing upward looking transducers of the scientific echo sounder (38 and 120 kHz) in front of the research vessel. The efficiency of the new approach for monitoring juvenile fish at night was investigated by comparing the results with a quantitative fry trawl in the Římov Reservoir in the Czech Republic. The experimental setup enabled comparisons for the 0–3 m and 3–6 m depth layers, which are utilized by almost all juvenile fish in summer. No statistically significant differences in the estimated abundance of juveniles were found between the two sampling methods. The comparison of abundance estimates gathered by the two frequencies were also not significantly different. The predicted mean lengths from acoustic sampling and the trawl catches differed by less than 10 mm in all comparisons. Results suggest that mobile hydroacoustic upward-looking systems can fill the methodological gap in non-invasive surveying of surface fishes.

## Introduction

Pelagic layers often represent the biggest volumes of large waterbodies. Surface layers (epilimnion) receive the most sunlight and are in contact with the atmosphere^1^. Very often, it is the most productive layer of the water column, and unlike the deeper layers, contains most phytoplankton, zooplankton and fish^2, 3^. Freshwater fish dominance near the water surface mainly at night can be found almost everywhere in the world, for example, Europe (The Czech republic, Germany, France, Hungary, Norway, Poland, United Kingdom, Switzerland etc.)^4–11^, North and South America (United States of America, Canada, Argentina)^12–14^ or in tropical areas (Sri Lanka, Thailand)^15, 16^.

Because of their high abundance, juvenile fish play an essential role in food webs and can indicate the future development of the fish stock as a whole^17^. In lakes and reservoirs, juvenile fish often hide in littoral habitats or use benthic refugia during the day and spread to the open water at dusk to utilize pelagic food resources^5, 9, 18^. During the growing season, most fish use the upper layers in mesotrophic and eutrophic waters, which are the warmest, the most productive and unlike the deeper layers have no limits with respect to dissolved oxygen levels^4, 19^. Juvenile fish also occur in the upper layers of oligotrophic lakes at night to follow vertical migration of zooplankton and to reduce their vulnerability to piscivorous fish^13, 20, 21^. Up until now, the only established quantitative method to study small fish in the upper waters was night trawling^22^. However, this method is labour-intensive, disrupts fish in their environment and may injure or kill juvenile fish.

Hydroacoustic methods are becoming increasingly popular because they can sample large volumes of water relatively quickly, are non-invasive and are non-lethal to the aquatic organisms being studied. The most commonly used acoustic approach is using downward-looking transducers, which beam from the surface to the bottom (i.e. from a boat or other platform to the deepest point). However, there is a blind zone at the surface created by the depth of the deployed transducer (at least several cm) and the physical near-field where the acoustic beam is not fully formed^23^. Additionally, near the transducer, the sampling volume is very low and provides a very limited coverage of the near-surface layers. In stratified lakes, fish often occur only a few centimetres to a few metres under the surface and, for this reason, the downward-looking approach does not provide reliable data near the surface and is often replaced by horizontal echo sounding^24, 25^.

Horizontal echo sounding, also called horizontal beaming, covers the surface layers well but has several major shortcomings. The most critical problem is that the estimated size of fish changes at different orientations relative to the transducer axis, the so-called side aspect^26, 27^. The determination of abundance and size is often performed using deconvolution^25^, which is based on stochastic assumptions of random aspect orientation that may not be entirely true^28^. Moreover, upon the establishment of thermal stratification, the acoustic beam can bend due to the effect of water temperature on the speed of sound on the edge of beam at different temperature layers^29^, thus complicating the definition of sampled volume. New findings on the multipath signals in the horizontal beam show strong interference near the surface^30^. Higher acoustic noise levels due to reverberation also complicate the detection of mainly small fish with horizontal beaming^28^.

A potential solution to address the aforementioned disadvantages of both downward-looking and horizontal beaming is an upward-looking system, where the transducer is oriented vertically, but the direction of beaming is from the water column towards the surface. This arrangement makes it possible to record fish in the near surface layer and to accurately determine their size. So far, this type of system is mostly restricted to stationary locations where the transducer is fixed to the bottom of the water body and continually samples the same volume^31–33^. One of the earliest mobile uses of an upward-looking system was by Probst^34^ who used a towed upward-facing transducer to study juvenile fish. However, towed systems are very sensitive to direction change and reduce the manoeuvrability of the towing vessel; in their case, a radius of approximately 250 m was required to change the direction of the vessel. It is also possible to use Remotely Operated Vehicles^35^, but their costs and the risk of collision with irregular bottom are still high. To circumvent these disadvantages, we developed a rigid upward-looking system located in front of the survey vessel to overcome all the sampling shortcomings described above. The aim of this study was to compare difference in abundance and size distribution between a new mobile upward-looking acoustic system and quantitative night fry trawling. On the acoustic side of the experiment we employed a frequency commonly used to detect small fish with narrow beam (120 kHz) side by side with the frequency mostly used for large fish with wider beam (38 kHz). Pros and cons of the two approaches are being compared.

## Results

The number of fish captured in the two sampled layers depends on the true depth distribution of fish (Table 1). Based upon trawling the number of fish in the 0–3 m layer was about eight times higher than in the 3–6 m layer. With acoustic data this ratio is even higher due to the beam morphology which is wider in the shallowest layer (larger sampling volume, Table 2). During one cruise the acoustic wedge volume was smaller than the volume sampled by the 3 × 3 m trawl. However, the echosounder sampled both layers at a time while the trawl only sampled one. The volume sampled by the 120 kHz system was considerably smaller due to its smaller beam dimension and the fact that it was only used during two sampling nights.

**Table 1:** Numbers of acoustically detected fish tracks and fish captured by the fry trawl.

survey cruise	depth 0–3 m	depth 3–6 m
38 kHz (night 1–3)	390/497/550	39/82/43
120 kHz (night 2 and 3)	437/704	15/21
Fry trawl (night 1–3)	488/1096/550	24/164/93

The results for individual nights are separated by /.

**Table 2:** Sampling volume of each cruise.

Sampling volume (m^3^)	depth 0–3 m				depth 3–6 m			
	Upstream		Downstream		Downstream		Upstream	
	38 kHz	120 kHz^a^	38 kHz	120 kHz^a^	38 kHz	120 kHz^a^	38 kHz	120 kHz^a^
Counted as wedge	52,700	17,300	54,800	18,400	33,700	11,200	33,800	9,200
Trawl	85,500	85,500	NA	NA	74,300	74,300	NA	NA

^a^Only night 2 and 3, NA – not applicable.

The dominant trawling catch species in the 0–3 m depth layer were roach (*Rutilus rutilus*), common bream (*Abramis brama*) and bleak (*Alburnus alburnus*) while perch (*Perca fluviatilis*), pikeperch (*Sander lucioperca*) and bream dominated in the 3–6 m depth layer (Supplementary Table S2).

### Fish length frequency distribution

**Figure 1:**
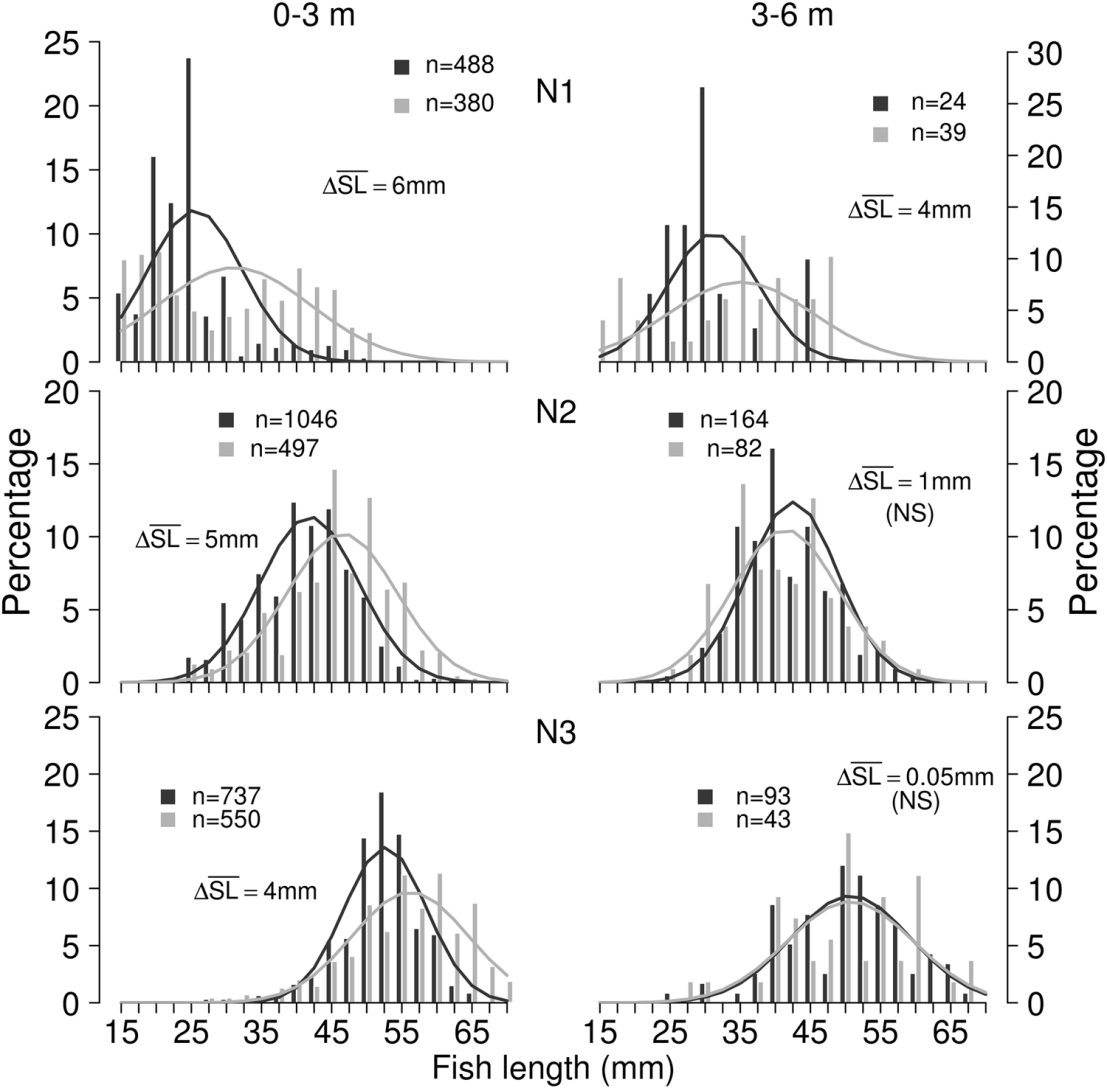
Length frequency distribution of juvenile fish on each night. Numbers of fish in the trawl catches (black) and tracked fish in the 38 kHz acoustic records (grey) are provided for each night and each depth layer. ΔTL stands for the difference between average acoustic and trawl length. NS – nonsignificant difference.

The new hydroacoustic system was able to record many sizes of fish (Supplement Fig. S1). Selecting a lower threshold was difficult on night 1 (19/20 August 2013) because of the high occurrence of newly-hatched fry measuring 15–25 mm (Fig. 1). For 2014 data it was easier to define troughs in the size frequency distributions because the YOY fish were considerably larger (30–50 mm).

When all YOY fish lengths were compared using both methods (upward-looking hydroacoustic system at a 38 kHz frequency and fry trawl), the observed size overlap was generally high (Fig. 1). The size distributions differences were statistically significant from zero for the 0–3 m depth layer but mostly not significant for the 3–6 m depth layer (Table 3). When comparing the length frequency distribution on different nights, we found that mean length of juvenile fish in the trawl were significantly smaller than from the hydroacoustic method in the 0–3 m layer, (night 1:6 mm mean difference, night 2 and 3:5 and 4 mm mean difference respectively; Fig. 1). In the 3–6 m layer the mean length of juvenile fish was significantly smaller in the trawl only in one case (night 1:4 mm mean difference, other nights’ sample the differences were negligible, Fig. 1, Table 3).

**Table 3:** Statistical results for juvenile abundance and size comparison between methods.

Compare	Test	Df	p-value*
0–3 m slope = 1	slope estimate	15	< 0.001
3–6 m slope = 1	slope estimate	12	>0.025
0–3 m abundance	Ks.test	17;17	>0.025
3–6 m abundance	Ks.test	14;14	>0.025
0–3 m abundance - upstream and downstream cruise	Ks.test	17;17	>0.025
3–6 m abundance - downstream and upstream cruise	Ks.test	17;17	>0.025
N1_0–3 m length dist.	Ks.test	488; 380	<0.008
N1_3–6 m length dist.	Ks.test	24; 39	<0.008
N2_0–3 m length dist.	Ks.test	1046; 497	<0.008
N2_3–6 m length dist.	Ks.test	164; 82	>0.008
N3_0–3 m length dist.	Ks.test	737; 550	<0.008
N3_3–6 m length dist.	Ks.test	93; 43	>0.008
N2_0–3 m length dist. 120 kHz	Ks.test	1046;437	<0.013
N2_3–6 m length dist. 120 kHz	Ks.test	164;15	<0.001
N3_0–3 m length dist. 120 kHz	Ks.test	737;704	<0.001
N3_3–6 m length dist. 120 kHz	Ks.test	93;21	<0.001

N1, N2 and N3 refer for night 1, night 2 and night 3, Ks.test refers to Kolmogorov – Smirnov test, dist. – distribution, *Bonferroni correction (0.025, 0.0125, 0.0083, respectively 2, 4, 6 same test).

**Figure 2:**
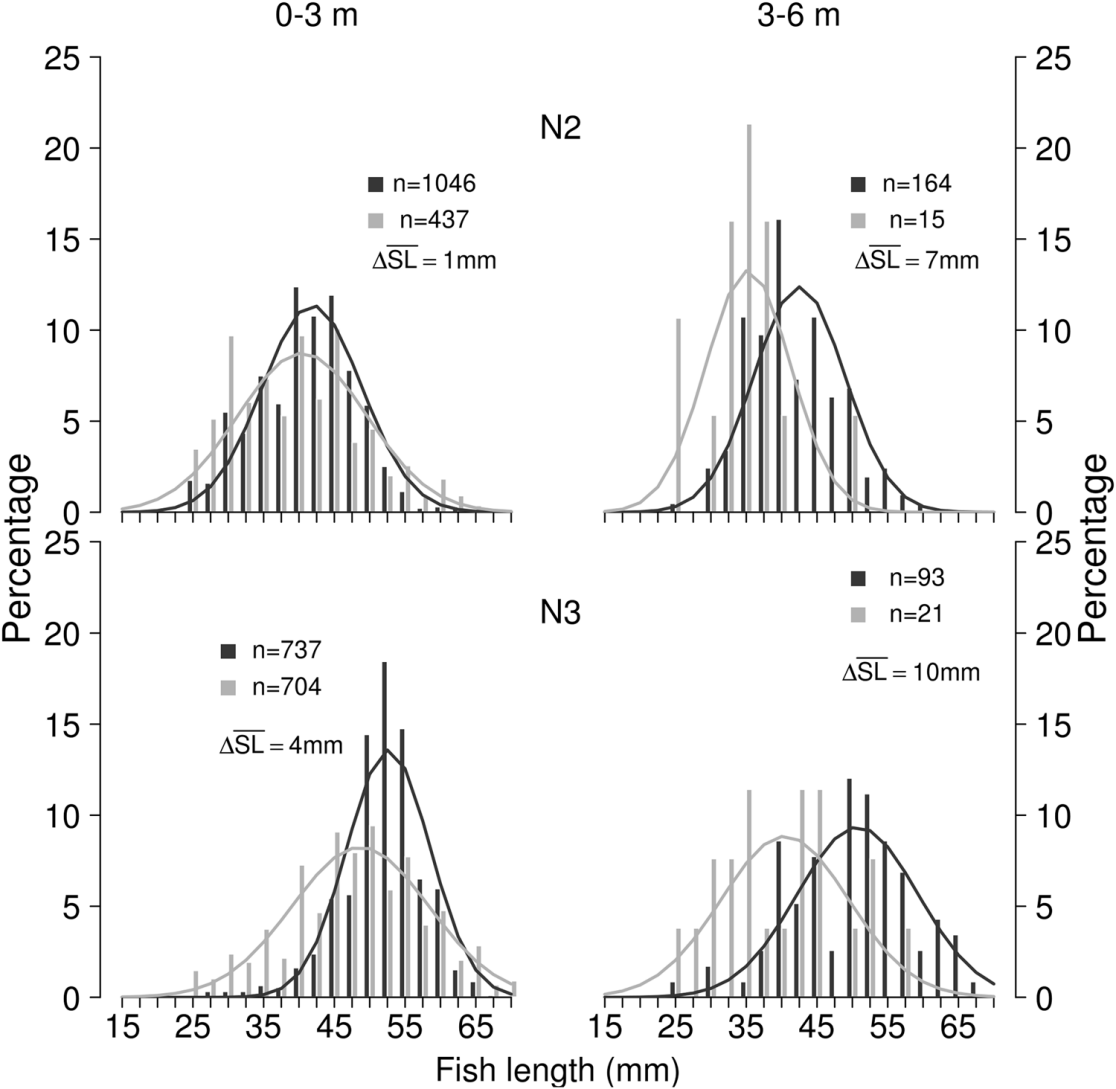
Length frequency distribution of fish from 120 kHz echo sounder and trawling. Numbers of fish in the trawl catches (black) and tracked fish in the 120 kHz acoustic records (grey) are provided for each night and each depth layer. ΔTL stands for the difference between average acoustic and trawl length.

In 2014, the juvenile pelagic community was also investigated using a 120 kHz echo sounder. When comparing the length frequency distribution by the trawl to the one reconstructed from the 120 kHz frequency acoustic results we can see reasonable overlap again. The difference between the trawl and the acoustic results was quite similar to that obtained with the 38 kHz system (acoustics record bigger fish in night 2:1 mm and night 3:4 mm, for depth 0–3 m and d 7 and 10 mm for 3–6 m, with the limitation of smaller numbers, Fig. 2). Despite the high overlap of the two distributions, the differences were statistically significant.

### Fish abundance

**Figure 3:**
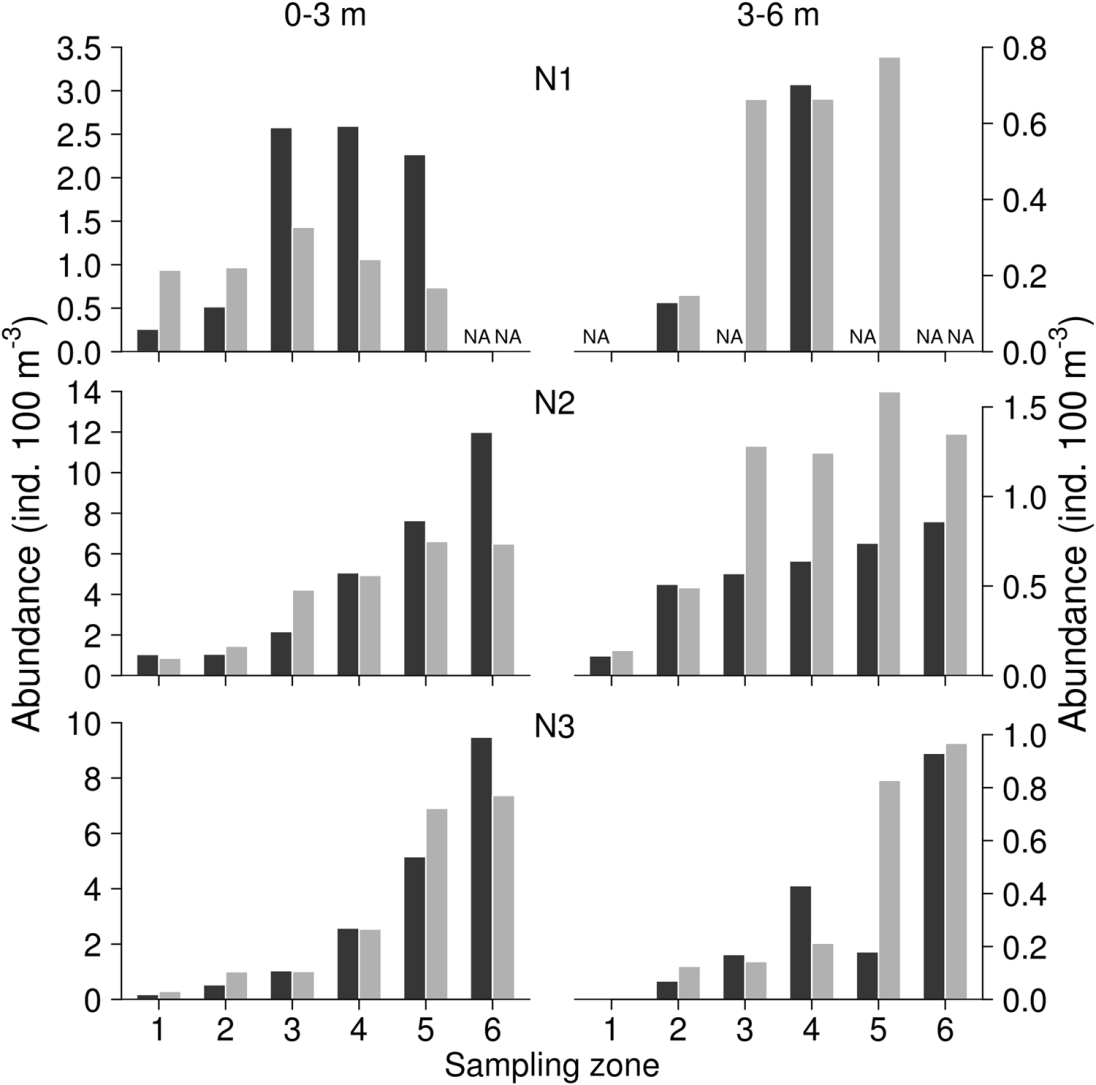
Abundance of juvenile fish on each night (N1–N3, 38 kHz). In each zone (1–6) and in 0–3 m (upstream cruise) and 3–6 m (downstream cruise) sampled by trawling (black) and upward-looking (grey). NA – not sampled.

**Figure 4:**
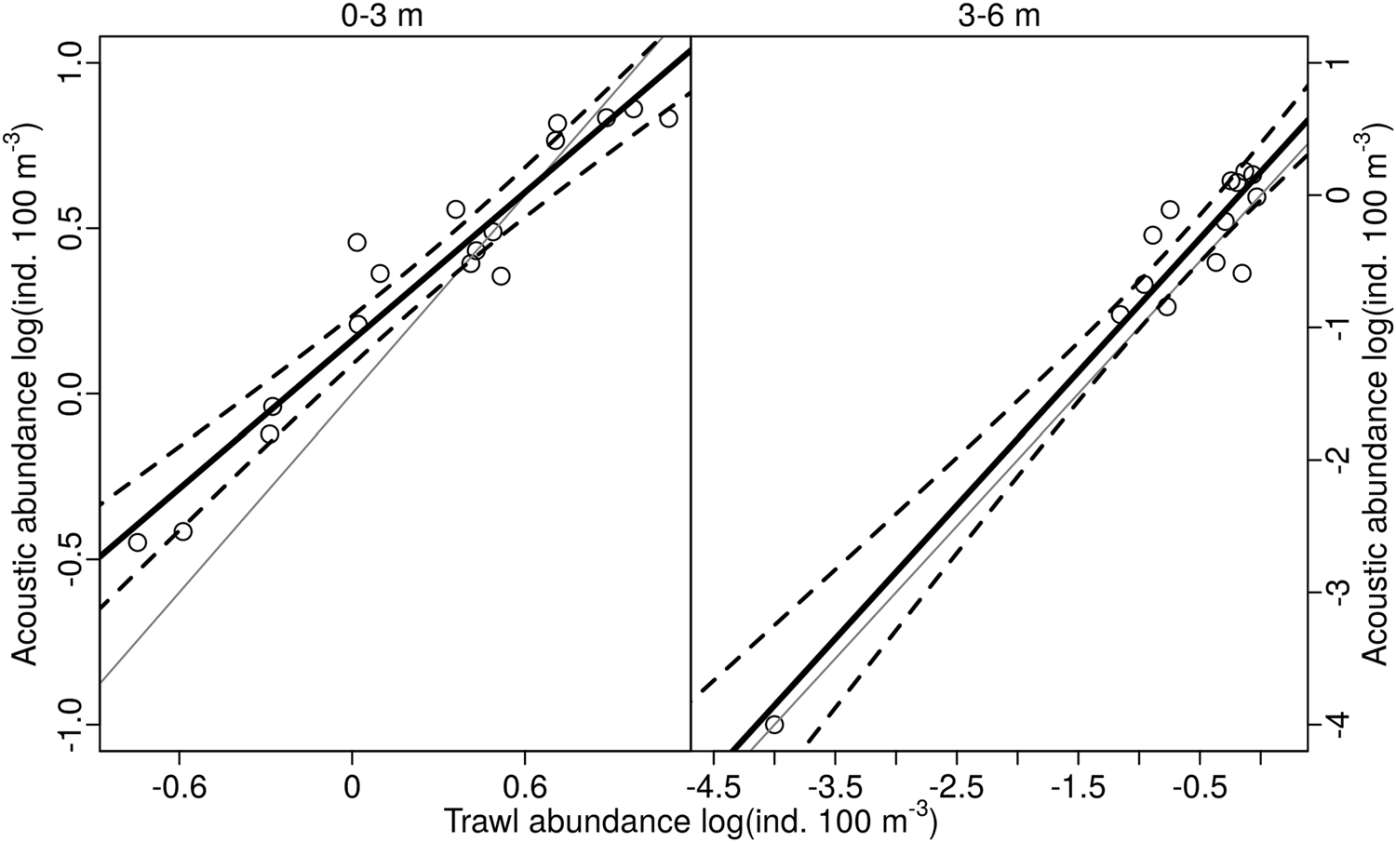
Linear regression model between upward-looking hydroacoustic abundance and trawl abundance (38 kHz). Each dot represents a separate sampling event. The regression line is displayed in solid black and the 95% confidence intervals are displayed in dotted lines. The 1:1 line is displayed in solid grey. The regression equation for each depth layer (y = 0.092 + 0.691x; r^2^ = 0.75 and y = 0.15 + 1.029x; r^2^ = 0.92, respectively for the 0–3 m and 3–6 m depth layers).

In the 0–3 m depth layer, abundance estimates obtained using both methods (acoustic frequency 38 kHz and trawl) in all zones during all sampling dates showed similar trends with lower density in the dam area (Fig. 3), and no significant differences were found between both methods (Table 3). Abundances estimated by acoustics and trawl were close to 1:1 line but in zones with higher densities of fish, the acoustic track counting tended to underestimate the abundance in the 0–3 m depth layer (Fig. 4). In the 3–6 m depth layer, differences between the two sampling methods were more common (Fig. 1 and Table 2), but in general, the slope of the relationship was not different from the 1:1 line (Fig. 4 and Table 3).

**Figure 5:**
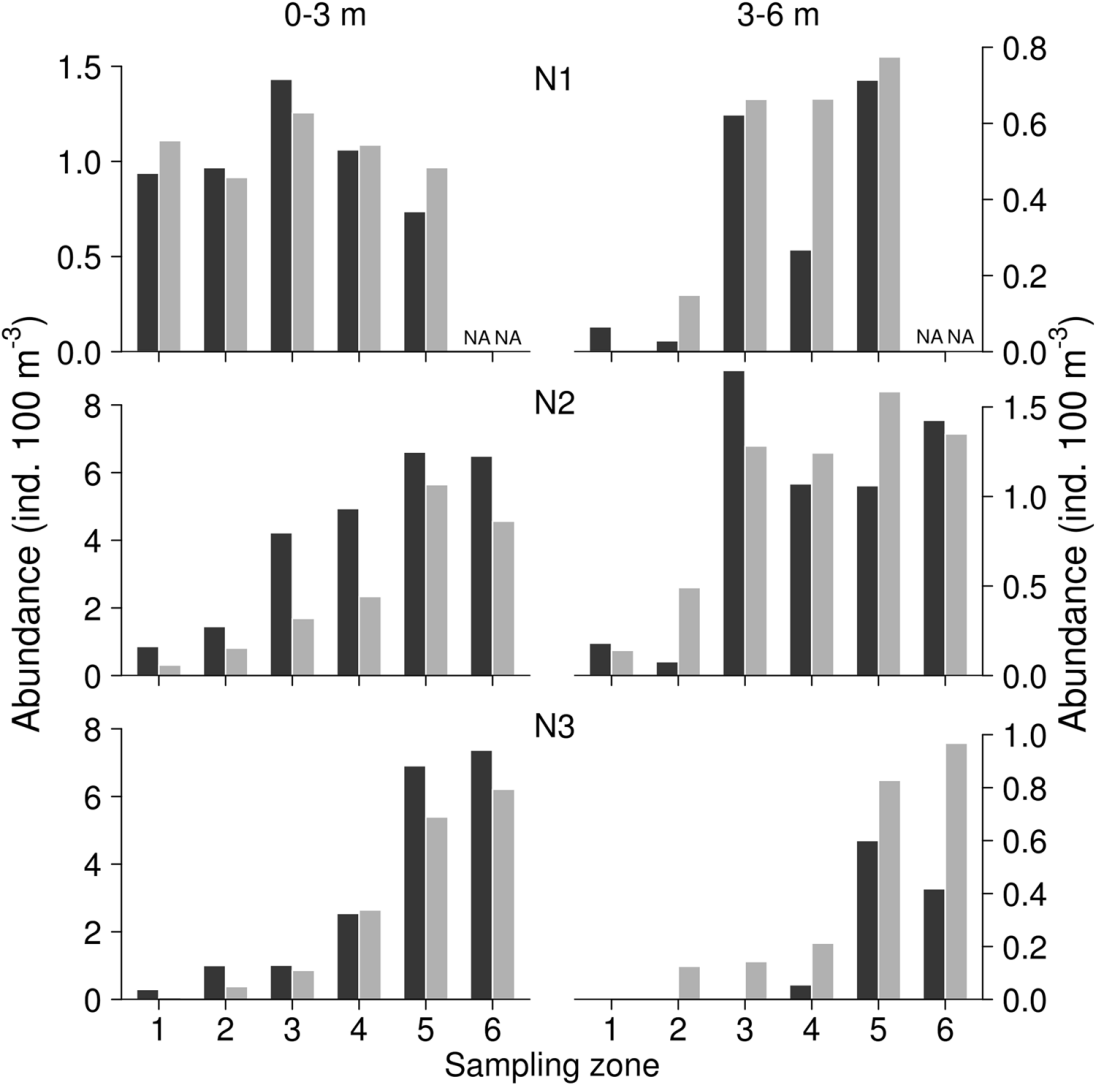
Replicability of abundance results of the two subsequent acoustic surveys (38 kHz). Abundance estimates obtained from the upward-looking hydroacoustic system during the three night of sampling (N1, N2 and N3) in each of the six sampling zones and in both the 0–3 m (left column) and 3–6 m (right column) depth layers. The estimates obtained during the upstream cruise are shown in black and those obtained during the downstream cruise are shown in grey. NB: in 3–6 m layer occasionally no fish of relevant size occurred. NA – not sampled.

Upstream and downstream acoustic cruises again showed similar trends in fish abundance (Fig. 5). No statistical difference in abundance was found between these two cruises (Table 3).

### TS distribution

Comparison of TS distribution from the two acoustic frequencies showed a rather similar pattern especially in N3 depth of 0–3 m (Supplementary Fig. S2). However, TS distribution was still significantly different (KS test p < 0.001, for N2 and N3 in depth 0–3 m). TS frequency distributions in depth 3–6 m were not significantly different (KS test p > 0.05). Fish length predicted from 120 kHz records were on average several mm smaller than from 38 kHz records (Supplementary Fig. S6) and therefore they are a bit closer to the sizes of trawl caught fish (Figs 1 and 2).

## Discussion

Results show that our novel upward-looking system and fry trawling provided comparable estimates of YOY fish abundance, as well as similar size distributions. Acoustic detection of small fish is to a great extent a question of a) signal to noise ratio (SNR) and b) of “linearity” in the regression function between log fish length and TS toward the lower end of the fish size spectrum^36^. When sampling a wind protected lake on calm nights at short ranges 0–6 m, the acoustic environment was extremely clean (noise level <−75 dB). The system could reliably detect the smallest fish in the water column at both frequencies tested.

The majority of acoustic data were collected with a 38 kHz transducer, a device that is scarcely used in freshwater systems. The most important reason for applying this frequency was the transducer’s opening angle of 12 degrees, which was, the widest split-beam transducer available at the time. Because even wider 38 kHz transducers are developed (Frank Knudsen, Kongsberg – Simrad, personal communication), these kinds of transducers are very promising for upward looking applications in the near future. The sampling volume when compared to the common 7-degree transducer is about three times larger (Table 2). 38 kHz has not been tested extensively for detecting small fish. That we could see fish well with this low frequency can be a bit surprising since theory states that targets should be generally bigger than the wavelength^36^. For 38 kHz the wavelength is about 40 mm, which is more than double the length of the commonly observed 15 mm fish, and much longer than the swimbladder for these fishes. Much of the theory describing this is, however, related to solid rigid spheres stating that targets smaller than the wavelength are in the Rayleigh scatter zone^37^. Fish are, however, not fixed, rigid, spheres. The swimbladder is shaped more like an ellipsoid than a sphere and according to Medwin *et al*.^38^ we can calculate a radius for an equivalent sphere. Doing this for fish as small as 15 mm, we find that we stay well within the safe zone for detecting small fish with the 38 kHz transducer^38^. We also did a trial with a 120 kHz system running in parallel with the 38 kHz system and verified that the two systems gave similar results. When Love^39^ did his work on TS regression for 38 kHz he also included fish down to 15 mm, and reported detecting them without problems.

The mean lengths of trawl-caught fish and those predicted by the upward-looking method differed by less than 10 mm. In all synoptic comparisons fish sizes predicted from acoustic data usually have a wider spread (higher variance) when compared with direct catch measurements^40–42^. Our results show this trend only to a small extent (Fig. 1). The largest potential source of error for predicting fish size from acoustic records is the TS-length regression. We have used two published regressions available to predict length. One was 38 kHz general multispecies regression for a dorsal aspect^39^ and the other 120 kHz regression for perch^43^. Several millimetres differences between predicted lengths for acoustic sampling and trawl-caught fish lengths (Figs 1 and 2) supports the need for more aspect-, frequency- and species-specific regressions between TS and fish size in the future. More attention should be paid to the experimental conditions (free swimming fish should be preferred to tethered and stunned individuals) which could influence the regressions^23^. The tank experiments of free-swimming larvae that would include repeated measurements of larvae and juveniles as they grow through time would be the ideal way to develop better models relating TS to length.

We did not find any significant differences in the abundance estimates obtained from the acoustic method and from the trawl. Furthermore, the regression curve correlating them was not significantly different from 1:1 in the 3–6 m depth layer. However, we have found that the slope was significantly different from 1:1 in the 0–3 m depth layer. This difference was probably caused by high densities of fish that were not recognized as single targets in the acoustic tracks. This result can be caused by overlapping echoes that do not satisfy SED criteria and cannot be tracked^23, 44^. So even at relatively small sampling volumes, there may be a need to use echo integration in addition to single target analysis to estimate the total abundance in dense fish communities. This is supported by higher values of Sawada index in some observations (Supplementary Table S3)^45^. Echo integration gave abundance estimate that were higher but not significantly different from the trawl (Supplementary Fig. S3, KS. test p > 0.05 for both depth). However with echo integration of small targets another challenge emerges with the need of laborious and potentially subjective removing of fish larger than the targeted YOY group^46^. Therefore, we consider the track counting to be generally more accurate for estimating larval fish abundance.

Although fish distribution had a relatively simple longitudinal pattern in the Římov reservoir (steady increase in fish abundance from the dam towards the tributary, Figs 3 and 5), this pattern was not identified over all surveys. The discrepancies between hydroacoustics and trawling results can be caused by the fact that despite the two sampling boats following very similar trajectories, the trajectories were not completely identical in space and time. Disturbing effect of passing boats during the survey at night is less likely^22, 47, 48^, but cannot be excluded completely^49^.

As described in the introduction, horizontal beaming has a number of limitations and also does not provide reliable estimates of juvenile fish. Mobile upward-looking hydroacoustic transducers largely overcomes the drawbacks of both horizontal beaming and downward-looking transducers when the goal is to survey the upper layer of a water body. In addition, the results obtained using our mobile upward-looking method were comparable with quantitative juvenile trawling. Still, the mobile upward-looking method has two obvious limitations:

1. The presented set-up has two 12 m long holding arms that can only be used on relatively large research vessels (Fig. 6). Nevertheless, it is possible to reduce the length of the arms if surveys were limited to shallower layers. In such case, it would be possible to use the equipment in shallower lakes but with the trade-off of significantly reduced sampling volume. Potential larger sampling volumes can be achieved by using a transducer with a wider beam (transducers with a wider are now being sold (Frank Knudsen, Simrad Inc., Kongsberg – personal communication).

**Figure 6:**
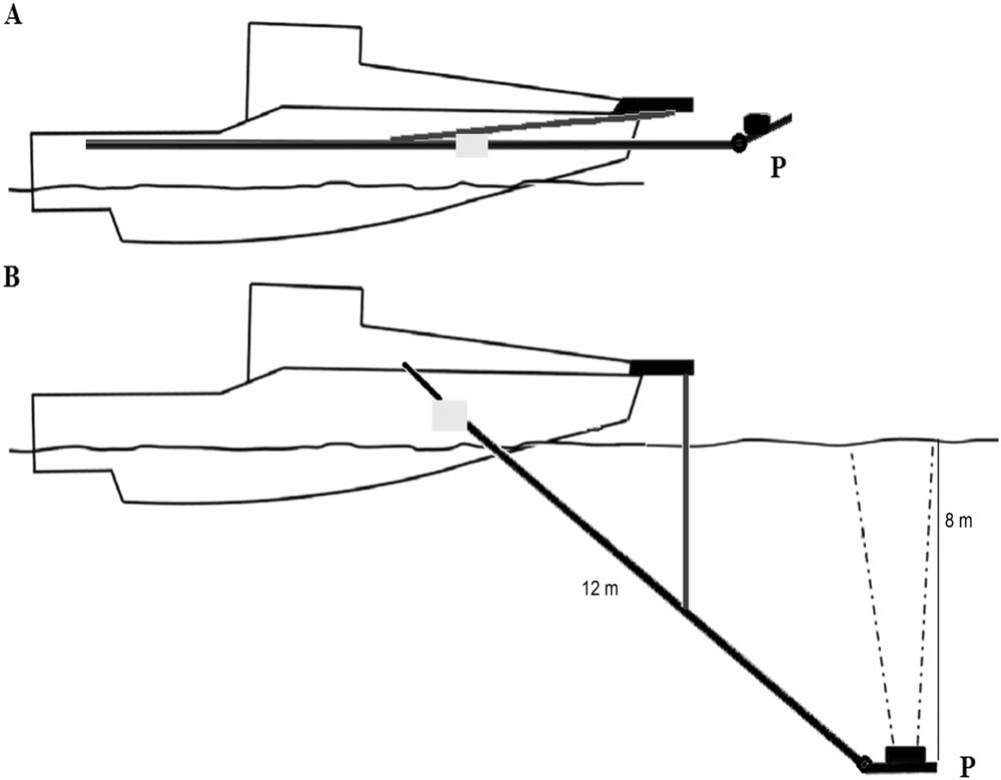
Schematic representation of the upward-looking acoustic system. (**A**) transport position and (**B**) operational position. P - tilltable platform holding the transducers, see details^51^.
2. Currently, our results are only for juvenile fish. Our acoustic records indicated many larger fish present in our studied reservoirs (Supplementary Fig. S1). However, no real-time capture methods for larger fish were available during this study such as pelagic trawling with a large trawl. The direct suitability of the proposed mobile upward-looking hydroacoustic method for studying the yearling and older portion of the fish stock should be verified in future studies.

The new sampling method circumvents the known disadvantages of horizontal and downward-looking hydroacoustic transducers when sampling above the thermocline. It is possible to enumerate late summer larval and juvenile fish community with both 38 and 120 kHz acoustic systems. Mobile upward-looking hydroacoustics is a promising fish-friendly method for further quantitative studies of pelagic upper layer fish communities, which are of great importance in many aquatic ecosystems where fish inhabit productive surface layers.

## Material and Method

### Study area

**Figure 7:**
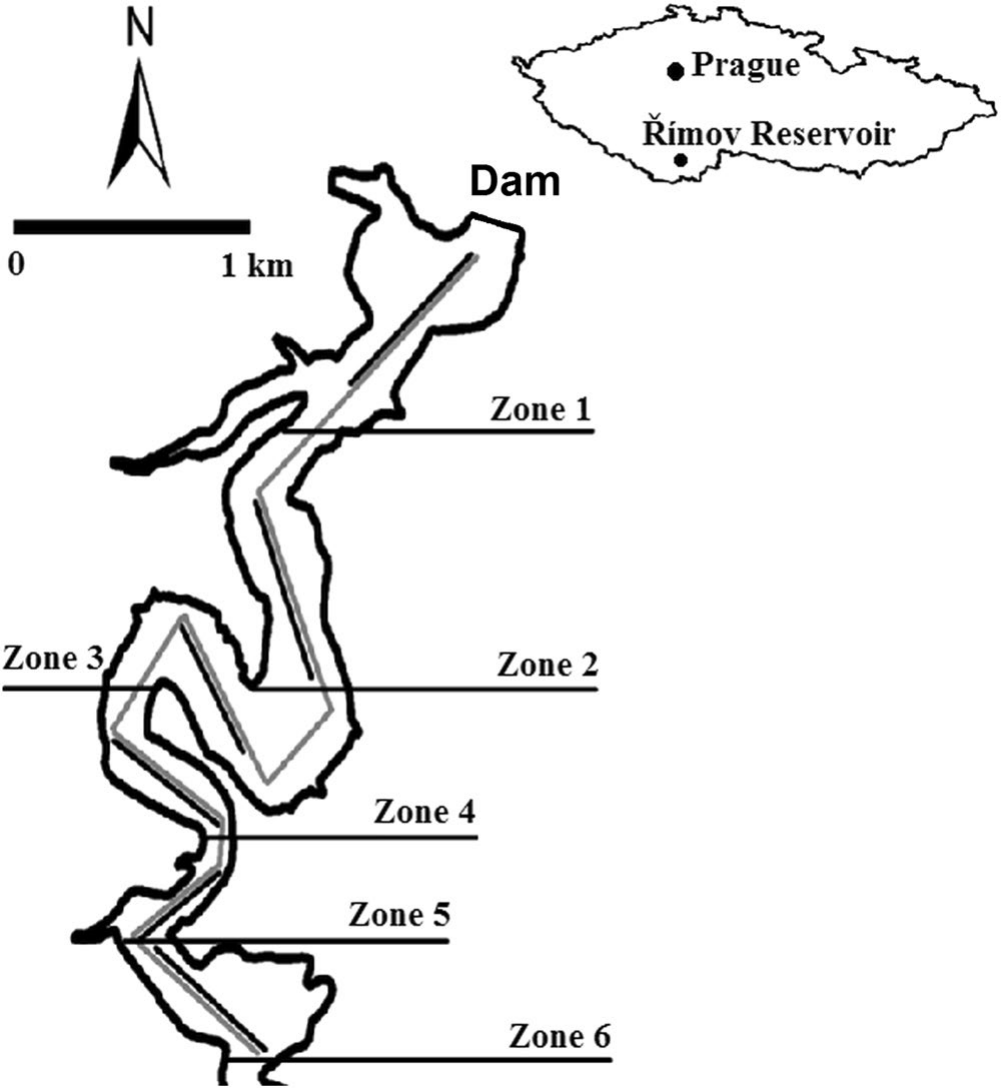
A map of the Římov Reservoir and location in the Czech Republic. The grey line shows the trajectory of the mobile upward-looking survey, and the black line indicates trawl sampling. Six sampled zones are also displayed (indicator lines show the southern end of the sampled zone). Figure was created by ArcMap 10.2.

This study was conducted in the Římov Reservoir, Czech Republic (48°50′N, 19°30′E, 471 m a.s.l., Fig. 7), which was constructed on the Malše River in 1978. It is a canyon-shaped reservoir with a total length of 12 km, a maximum volume of 33 × 10^6^ m^3^, a surface area of 2.1 km^2^, and an average and maximum depth of 16 m and 45 m, respectively. The trophic state of the reservoir is mesotrophic to eutrophic with the dominance of common bream, roach and bleak in both juvenile and mature fish communities^19, 50^. Due to the strong temperature and oxygen vertical gradients during summer months, both juvenile and adult fish inhabit the water layer above the thermocline^4, 19, 32^.

Vertical profiles of temperature and dissolved oxygen were measured during all surveys (Supplementary S4) from the surface to 10 m depth in zone 1 using a calibrated thermistor YSI 556 MPS probe. Surveys were performed on 19/20 August 2013 (night 1), 23/24 July 2014 (night 2) and 8/9 August 2014 (night 3).

### Acoustic system

The acoustic part of the study was executed using a newly-developed method based on a mobile upward-looking acoustic system^51^. To implement this approach, an epoxide laminate research vessel 11 m long and 3 m wide with a 210 HP engine was equipped with two 12 m lifting arms on either side that held a platform with adjustable tilt and transducer(s) (Fig. 6). During the acoustic survey, the lifting arms submerged the platform to a depth of 8 m, approximately 5 m in front of the vessel. The platform was tilted so that the transducer faced the surface and was beaming upwards perpendicularly (the exact vertical position of the acoustic beam was verified using an electronic clinometer RIEKER H5A1-90).

Acoustic measurements were collected using primarily a Simrad EK60 split-beam echo sounder operating at a frequency of 38 kHz (circular transducer SIMRAD ES38-12 with a nominal angle of 12 deg.). This echo sounder was chosen for three main reasons. First, this frequency has minimal sensitivity to aquatic invertebrates such as *Chaoborus* larvae, which could potentially interfere with small fish echoes^52^. The second reason was the need to maximize the sampling volume at short range. The Simrad ES38-12 transducer employed had the widest opening angle of all split-beam transducers that were commercially available at the time of the survey. Thirdly, 38 kHz transducer is the relatively low sensitivity of TS to small changes in fish tilt and the generally smaller TS variability of 38 kHz compared with higher frequencies^23^. In 2014 an additional echo sounder operating at a frequency of 120 kHz (circular split-beam transducer SIMRAD ES120-7G with a nominal angle of 7 deg.) was also used on the same platform. The operating power of the 38 kHz echo sounder was set to 100 W with 0.05 ms pulse interval (20 ping s^−1^) and the pulse length was set to 256 μs. The 120 kHz EK 60 echo sounder was set to 100 W with 0.05 ms pulse interval (20 ping s^−1^) and the pulse length was set to 128 μs (higher frequency has sufficient number of waves in shorter pulse allowing thus for higher spatial resolution and shorter blind zone). Before each survey, both transducers were calibrated in down looking position of the platform using a 60 mm diameter copper sphere for 38 kHz and 33.8 tungsten sphere for 120 kHz as per methods described by Foote^53^.

Raw acoustic data were converted and analysed using the Sonar5Pro post-processing software (Lindem Data Acquisition, Oslo, Norway). Beyond the theoretical blind zone with transducer ringing signal (half of the pulse width, 19 cm for 38 kHz, 8 cm for 120 kHz), we defined a safe margin of 0.1 m below so the surface echo was safely excluded from data processing prior to data analysis. The acoustic data were divided into two depth layers; to correspond to the layers sampled by the trawl (0.1–3 m, further called 0–3 m and 3–6 m below the surface). Data from both layers were recorded at the same time (Table 1).

Fish total length (TL) was calculated from target strength using regression parameters derived for various fish species at a 38 kHz frequency^39^ and for juvenile perch at a 120 kHz frequency^43^ (better agreement than^39^ for 120 kHz):

1 $${\rm{TL}}={10}^{(TS-\frac{63.38}{19.13})}\,{\rm{frequency}}\,{\rm{38}}\,{\rm{kHz}}$$

2 $${\rm{TL}}={10}^{(TS-\frac{86.41}{20.79})}\,{\rm{frequency}}\,{\rm{120}}\,{\rm{kHz}}$$

where TL is fish total length^39, 43^ in cm and TS is the target strength in dB.

Signal to noise ratio (SNR) can be seen by looking at the echoes from the smallest fish and compare them with background noise level composed of the background noise reverberation level and the echosounders electric noise. In our acoustic recorded data, we determined that the noise levels were lower than −75 dB. With the smallest fish targets having an off-axis compensated target strength of −60 dB we have an SNR of more than 15 dB for targets in the centre of the beam and still more than a 9 dB SNR for targets at the edge of the beam.

Acoustic surveys were performed in straight-line transects at a constant speed of 1 ms^−1^ following the original river valley (Fig. 7). To avoid striking the bottom with the submerged platform, only the deepest part (depth >10 m, 6 km long zone from the dam) of the reservoir was sampled (zone 1–6, Fig. 7). To determine the depth between the bottom and the platform, a downward-looking single-beam transducer (Simrad ES200 with nominal angle 9°) was mounted on the underside of the platform. Acoustic recordings started from the dam one hour after sunset (at approximately 22:30 p.m. in zone 1, Fig. 7) and finished in the middle part of the reservoir (finished approximately at 0:30 a.m. in zone 6, downstream, Supplementary Table S1). Recording was stopped at the end of zone 6, the boat was turned around and after a half hour waiting period (to avoid bubbles made by the boats propellers), the same transects were sampled in the opposite direction (from zone 6 towards zone 1 finished at approximately 3:45 a.m., upstream, Supplementary Table S1). The GPS location of the vessel was measured using a Garmin GPSMAP 60CSx GPS throughout the survey.

An automatic single echo detection (SED) primary threshold of −70 dB was used to define targets of interest. A fish track was defined as having at least three subsequent echoes of the same target, separated by a maximum of one missing ping within a 0.1 m vertical range. All tracks were manually checked and the tracks outside the nominal beam (between −6 and 6 or −3.5 and 3.5 degrees for 38 and 120 kHz system, respectively) were removed. The minimum and maximum acoustic thresholds for juvenile fish were set into the troughs of size frequency distributions^54^ accepting the targets of interest consistent with YOY fish. These limits were as follows for targets: TS −61.5 to −50 dB and corresponding to fish 15–50 mm in the trawl catch during night 1, (TS −55.8 to −47.8 dB and −55.7 to −45.60 dB, respectively for 38 and 120 kHz) corresponding to fish 25–65 mm in the trawl catch during night 2 and (TS −55.8 to −47.2 dB and −55.7 to −44.8 dB, respectively for 38 and 120 kHz) corresponding to fish 25–70 mm in the trawl catch during night 3.

Fish abundance was calculated according to the trace counting method^23, 54^:

3 $${({f/m}^{3})}_{{\rm{tracks}}}=tracks/{{\rm{v}}}_{{\rm{w}}}$$

where *tracks* stands for the number of tracks in a given transect and it is divided by the sampled wedge volume v_w_ in m^3^ (based on the equivalent beam angle and sailing distance). Fish abundance was reported as the number of fish in 100 m^3^ of sampled water (*f/100* *m* ^*3*^) for the 0–3 and 3–6 m depth layers.

The second method of Sv/TS scaling method was used to analyse recorded upward-looking data by echo integration (to compare with tracked fish densities, which can fail under high target densities). Echograms were analysed using the same threshold restrictivity as for track-counting. Fish bigger than −47.5 dB were erased by a special function of SONAR 5 software. In-situ fish tracks were used for estimating mean TS. Only targets of TS between −55.8 and −47.8 dB were used for night 2 and targets of TS between −55.8 and −47.2 dB were used for night 3.

### Direct fish sampling

Pelagic habitat sampling was performed using a 3 × 3 m fixed-frame fry trawl. The trawl body was 10.5 m long with a knot-to-knot mesh size of 6.5 mm in the main belly and 4 mm in the cod end (for details see ref. 22, Supplementary Fig. S5). The trawl was towed for 10 min approximately 100 m behind the second research vessel (trawler) at a speed of 1 ms^−1^. Samplings occurred at two depth layers (0–3, 3–6 m), the shallower depth was sampled during the upstream cruise and the deeper depth during the downstream one (Supplementary Table S1). During night 1, zone 6 was not sampled due to extremely low water levels in 2013. Trawling data from the layer 3–6 m from zones 1, 3 and 5 were also missing for this year. All trawling tows began approximately 20 minutes after the acoustic survey and had parallel trajectories (Fig. 7).

Fish caught by the trawl were immediately euthanized using a lethal dose of MS 222 and were subsequently preserved in a 4% formaldehyde solution. In the laboratory, fish were identified to the species level^55^, counted and TL was measured to the nearest mm. For each trawl tow, the sampled water volume was calculated based on the tow distance measured by GPS, and the CPUE (catch per unit effort) of the trawl tow was expressed as catch per 100 m^3^ of water sampled.

Animal treatment was performed under permission from the Experimental Animal Welfare Commission under the Ministry of Agriculture of the Czech Republic (Ref. No. CZ 01679). All methods were performed in accordance with project protocols approved by a named institutional and national committee (Ref. No. 77/2013).

### Statistical analyses

Differences in the abundance estimated by trawling and hydroacoustics sampling were compared using a Kolmogorov – Smirnov paired test for both depth layers separately. Additionally, the abundance estimates for both methods were regressed against each other, and we determined if the slope parameter was significantly different from unity^56^. Length frequency distributions of caught fish and predicted length frequency from tracked fish were tested by Kolmogorov – Smirnov paired for comparisons. Statistical analyses were carried out using the R language^57^.

## Electronic supplementary material

Supplementary information
