# Supplementary material for: A novel upward-looking hydroacoustic method for improving pelagic fish surveys
Source: Sci Rep. 2017 Jul 6;7:4823. doi: 10.1038/s41598-017-04953-6 (PMC5500586; doi:10.1038/s41598-017-04953-6)
Supplement: Supplementary file 1 — Supplementary information [file 41598_2017_4953_MOESM1_ESM.pdf]

Supplementary information for the paper:

A novel upward-looking hydroacoustic method for improving pelagic fish surveys.

By: Baran R., Jůza, T., Tušer, M., Balk, H., Blabolil P., Čech, M., Draštík, V., Frouzová, J., Jayasinghe A.D., Koliada, I., Mrkvička T., Muška, M., Ricard, D., Sajdlová, Z., Vejřík, L., Kubečka, J.

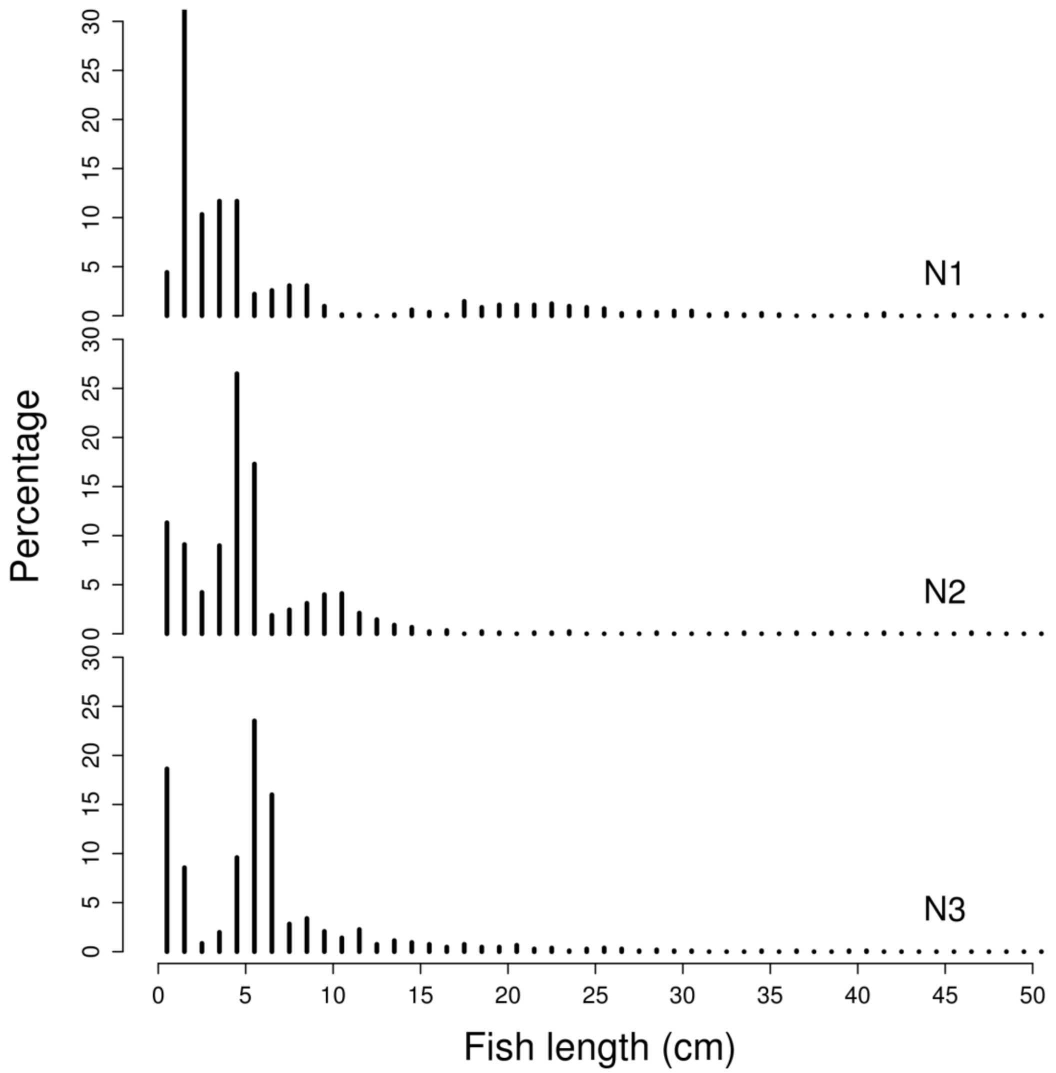

Supplementary Fig. S1. Length frequency distribution of all fish recorded by 38 kHz each night (N1-N3)

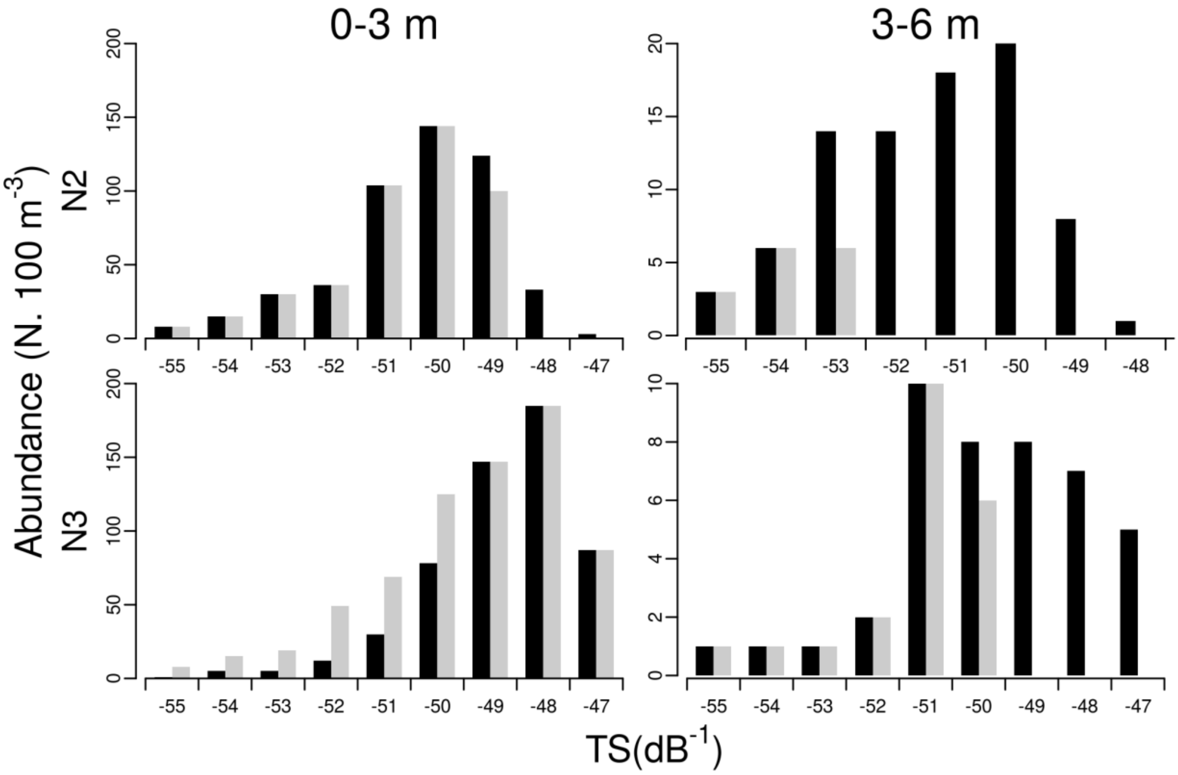

Supplementary Fig. S2. TS distribution of YOY fish records at both frequencies 38 and 120 kHz

TS distribution of frequency 38 kHz represents black and 120 kHz gray

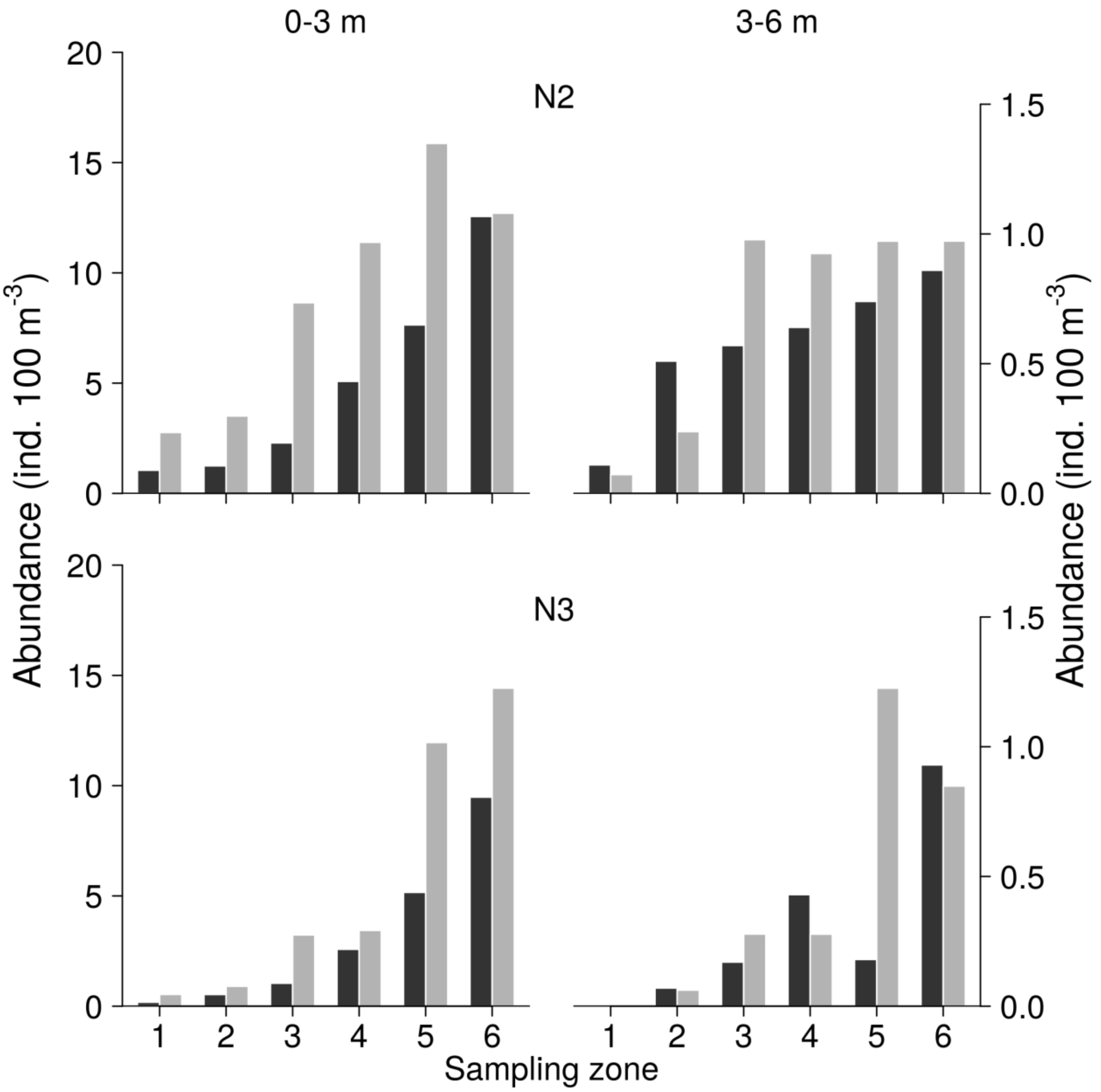

Supplementary Fig. S3. Compare echo integration with trawl abundance

In each zone (1-6) and in 0-3 m (upstream cruise) and 3-6 m (downstream cruise) sampled by trawling (black) and upward-looking echo integration (grey).

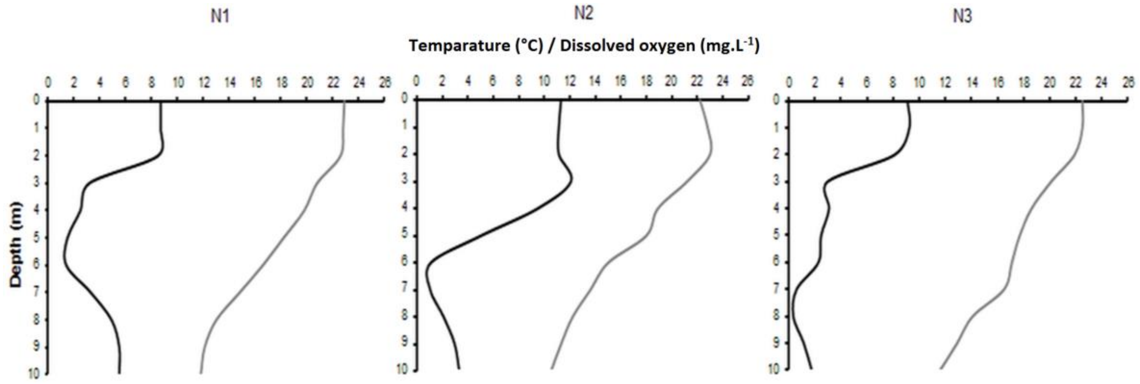

Supplementary Fig. S4. Vertical profile of water temperature.

Temperature (grey line) and dissolved oxygen (black line) measured in zone 1 immediately after the survey. N1, N2 and N3 refer to night 1, night 2 and night 3.

Supplementary Table S1. Survey design of acoustic and trawling data collections during the upstream and downstream cruises every night.

survey cruise	depth 0-3 m	depth 3-6 m
Upstream (from zone 1 to 6)	acoustic + fry trawl	acoustic
Downstream (from zone 6 to 1)	acoustic	acoustic + fry trawl

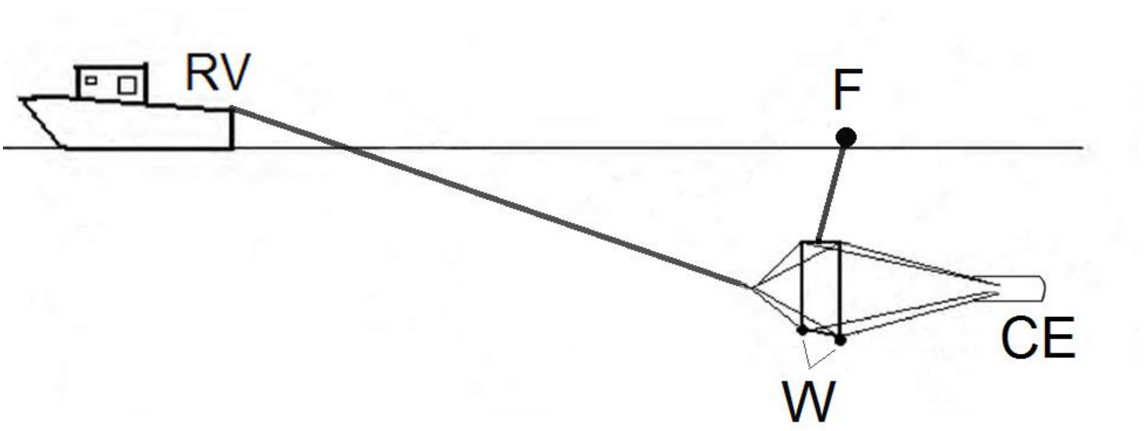

Supplementary Fig. S5. Schematic figure of trawl setting

RV – research vessel, F – floater, W – weights, CE – cod end

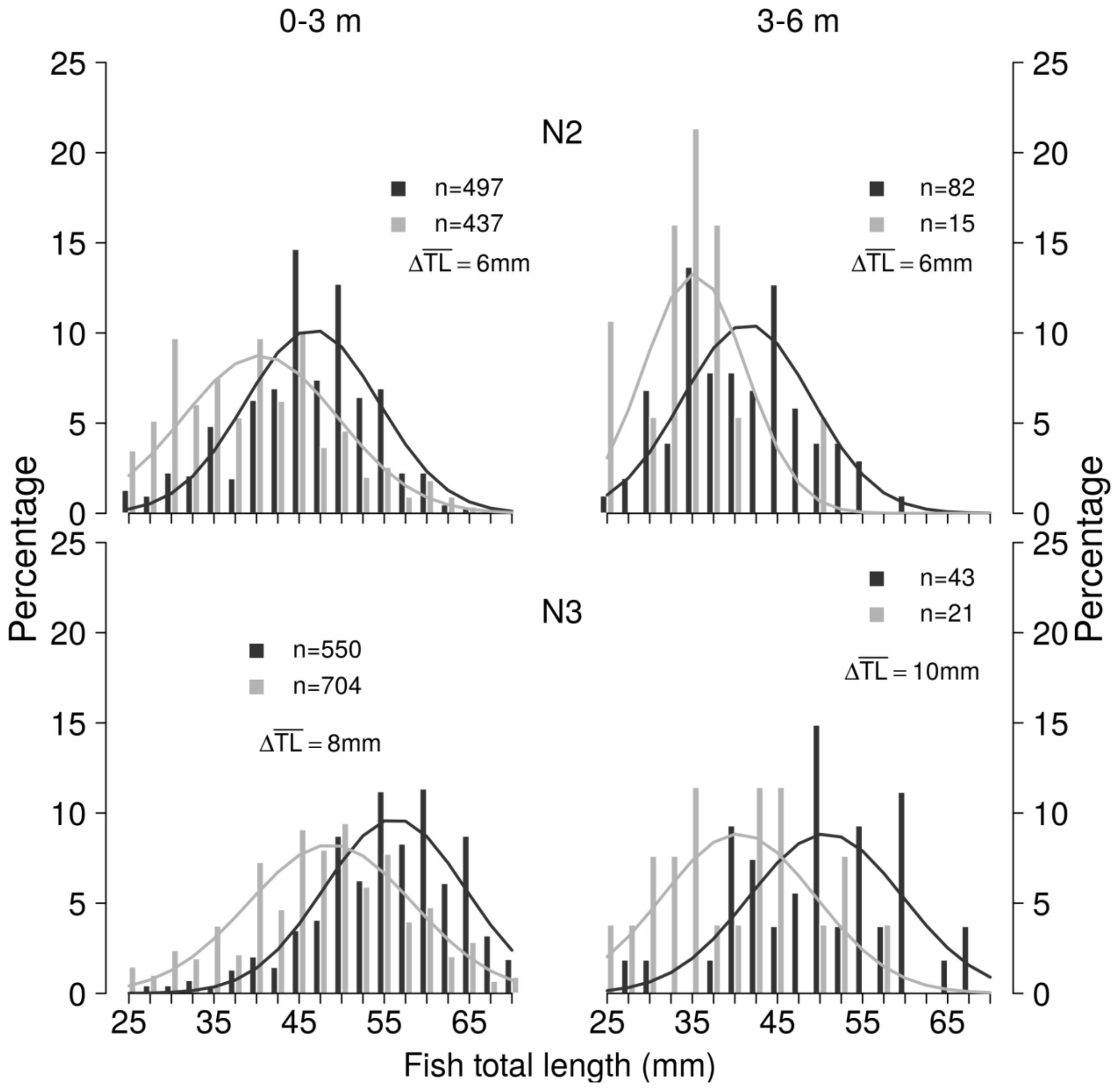

Supplementary Fig. S6. Length frequency distribution of YOY fish records in 38 and 120 kHz echo sounder

A number of tracked fish in the 120 kHz acoustic records (grey) and 38 kHz acoustic record (black) are provided for each night and each depth layer. ΔTL stands for the difference between average lengths estimated by the two frequences.

Supplementary Tab. S2. Percentual species composition of trawl catch in two depth layer.

		Depth 0-3 m			Depth 3-6 m		
**Species**	**Latin name**	**N1**	**N2**	**N3**	**N1**	**N2**	**N3**
Bream	Abramis brama	48.16	24.76	28.36	33.33	14.63	34.41
Roach	Rutilus rutilus	0.00	38.91	67.57	0.00	7.32	25.81
Bleak	Alburnus alburnus	19.26	11.57	3.26	4.17	0.61	1.08
Pikeperch	Sander lucioperca	20.90	7.74	0.54	20.83	15.24	11.83
Perch	Perca fluviatilis	2.05	14.63	0.14	4.17	60.98	25.81
ruffe	Gymnocephalus cernua	9.63	2.39	0.14	37.50	1.22	1.08

Supplementary Tab. S3. Sawada index for both acoustics frequencies

Zone	N2 0-3 m		N3 0-3 m		N2 3-6 m		N3 3-6 m	
	**38 kHz**	**120 kHz**	**38 kHz**	**120 kHz**	**38 kHz**	**120 kHz**	**38 kHz**	**120 kHz**
1	0.060	0.007	0.114	0.001	0.040	0.000	0.000	0.000
2	0.130	0.009	0.070	0.002	0.030	0.000	0.010	0.000
3	0.050	0.022	0.100	0.006	0.060	0.001	0.030	0.000
4	0.430	0.029	0.040	0.006	0.270	0.001	0.000	0.000
5	0.110	0.041	0.020	0.022	0.180	0.001	0.000	0.001
6	0.390	0.028	0.294	0.027	0.318	0.001	0.130	0.001
